# The multiple factors of suboptimal early feeding practices among infants aged 0–5 months in Indonesia

**DOI:** 10.3389/fnut.2023.1080727

**Published:** 2023-03-28

**Authors:** Christiana Rialine Titaley, Ratna U. Wijayanti, Anifatun Mu'asyaroh, Iwan Ariawan

**Affiliations:** ^1^Faculty of Medicine, Pattimura University, Poka Campus, Ambon, Indonesia; ^2^College of Health Science, Bhakti Pertiwi Indonesia, South Jakarta, Indonesia; ^3^UPTD Alian Health Center, District Health Office of Kebumen, Kebumen, Indonesia; ^4^Faculty of Public Health, Universitas Indonesia, Depok, West Java, Indonesia

**Keywords:** initiation of breastfeeding, prelacteal feeding, exclusive breastfeeding, early infant feeding practices, Indonesia demographic and health survey

## Abstract

**Background:**

Optimal early infant feeding practices are critical to ensure adequate nutrition for infants’ growth and development. This study aimed to examine the determinants of suboptimal early feeding practices (i.e., delayed initiation of breastfeeding, prelacteal feeding, and non-exclusive breastfeeding) among infants aged 0–5 months in Indonesia.

**Methods:**

We used data collected in the 2012 and 2017 Indonesia Demographic and Health Surveys. Analyses were conducted using information from 3,198 live-born singleton infants aged 0–5 months. The primary outcomes used were: (1) delayed initiation of breastfeeding in the first hour after birth, (2) prelacteal feeding in the first 3 days, and (3) non-exclusive breastfeeding in the last 24 h preceding the survey. Potential predictors analyzed were categorized into the environmental, household, maternal, pregnancy, delivery, and child characteristics. Logistic regression analyses were performed to identify factors significantly associated with each outcome.

**Results:**

Approximately 78,6% of infants aged 0–5 months in Indonesia had at least one of the three suboptimal early infant feeding practices. We found a strong association between the three outcome indicators analyzed. The determinants of delayed initiation of breastfeeding included infants from Sumatera region (adjusted odds ratios (aOR) = 2.02, *p* < 0.001), infants delivered by Cesarean section (aOR = 2.78, *p* < 0.001), and in non-health facilities (aOR = 1.53, *p* = 0.003). The determinants of prelacteal feeding in the first 3 days included infants living in urban areas (aOR = 1.32, *p =* 0.035), the first birth-ranked infants (aOR = 1.32, *p =* 0.019), and infants who had delayed initiation of breastfeeding in the first hour of life (aOR = 3.90, *p <* 0.001). The determinants of non-exclusive breastfeeding in the last 24 h included infants whose mothers worked in non-agricultural fields (aOR = 1.52, *p <* 0.001), infants delivered by Cesarean section (aOR = 1.33, *p =* 0.044), and the first birth-ranked infants (aOR = 1.28, *p =* 0.039).

**Conclusion:**

There was a high percentage of infants aged 0–5 months who had suboptimal feeding practices in Indonesia. As we found multiple factors associated with suboptimal early feeding practices among infants, integrated approaches, including health promotion and supportive public policy, are required to ensure infants receive adequate nutrition in the early stages of life.

## 1. Introduction

The first 2 years of life are fundamental for a child’s growth, development, as well as survival (1). Optimal early infant feeding practices, including early initiation of breastfeeding within 1 hour of birth, no-prelacteal feeds, and exclusive breastfeeding in the first 6 months of life, have vital roles in ensuring adequate nutrition in the early stages of infants’ life (2).

The benefits of early initiation of breastfeeding through mother-infant skin-to-skin contact in the first hour of life have been widely acknowledged (3, 4). Mother-infant skin-to-skin contact helps regulate newborn body temperature and allows infants to receive beneficial bacteria from the mother’s skin. (3).

One of the suboptimal feeding practices commonly reported in the early infancy period is the practice of prelacteal feeding, i.e., giving newborn food/liquid other than breastmilk, mostly in the first 3 days of life before commencing breastfeeding (5). This practice increases the risk of illness among newborns and prevents them from the protective effects and vital nutrients in the colostrum (6). Regardless of its detrimental effects on health, prelacteal feeding is frequently practiced worldwide, particularly in developing countries, including Indonesia (6, 7).

One of the most crucial feeding practices recommended by The World Health Organization (WHO) in the Global Strategy for Infant and Young Child Feeding is exclusive breastfeeding, defined as the practice of only giving infant breastmilk for the first 6 months of life (8). Infants who were exclusively breastfed had both long- and short-term protective effects, including reduced risk of infections, overweight, and obesity, as well as improved child survival (9–11). According to the WHO, only 44% of infants aged 0–5 months worldwide were exclusively breastfed between 2015 and 2020 (1). This was still below the Global Nutrition Target (at least 50% in 2025 and 70% in 2030) (12, 13).

In Indonesia, considerable progress has been made as the proportion of children exclusively breastfed increased from 32.4% in 2007 (14) to 52.0% in 2017 among children under 2 years old (15). Similarly, early initiation of breastfeeding also showed an increasing trend from 43.9% in 2007 (14) to 56.5% in 2017 (15). Although these numbers showed improved early infant feeding practices, efforts to accelerate them are still required.

Multiple factors have been linked to early initiation of breastfeeding and exclusive breastfeeding, including socioeconomic status (16), parental occupation (16, 17), parents’ knowledge and awareness (18), type of attendant at birth (19), place of delivery (16), mode of delivery (16, 19), child’s birth weight (19) and family supports (17). Another factor reported to deprive children of receiving valuable nutrients and protection from colostrum and also negatively associated with exclusive breastfeeding is the practice of prelacteal feeding (20).

Using nationally representative data from the 2012 and 2017 Indonesia Demographic and Health Surveys (IDHS), we examined the determinants of delayed breastfeeding initiation in the first hour of life, prelacteal feeding in the first 3 days of life, and non-exclusive breastfeeding practices in the last 24 h preceding each survey. Program managers and policymakers could use our findings to plan and implement programmatic actions to accelerate optimal breastfeeding practices in Indonesia, especially in the first 6 months of life.

## 2. Methods

### 2.1. Data source

We used data from the 2012 and 2017 IDHS, national surveys conducted regularly by Statistics Indonesia (*Badan Pusat Statistik*) in partnership with the National Population and Family Planning Board and the Ministry of Health of the Republic of Indonesia (15, 21). The IDHS aims to provide a comprehensive overview of population issues in Indonesia, particularly basic demographic and health indicators.

Four types of structured questionnaires were used in the 2012 and 2017 IDHS: Household, Woman, Currently Married Man, and Never-Married Man Questionnaire (15, 21). Information used in this analysis was derived from the Woman’s and Household’s Questionnaire. The Women’s Questionnaire collected basic demographic and health characteristics of women aged 15–49 years, including their background characteristics and antenatal, delivery, postnatal care, and breastfeeding practices. Detailed information about IDHS sampling methods and fieldwork management has been reported elsewhere (15, 21).

In the 2012 IDHS, a total of 43,852 households (99% response rate) and 45,607 eligible women (95.9% response rate) were interviewed, while in the 2017 IDHS, 47,963 households (99.5% response rates) and 49,627 eligible women (97.8% response rates) were interviewed (15, 21). For this analysis, we used information collected from 3,198 singleton live-born infants aged 0–5 months (1,621 from 2012 and 1,577 from the 2017 IDHS).

### 2.2. Study outcomes

There were three outcomes of suboptimal early infant feeding practices used in this analysis: (1) delayed breastfeeding initiation in the first hour of life, (2) prelacteal feeding in the first 3 days of life, and (3) non-exclusive breastfeeding in the last 24 h preceding the survey. The first outcome, initiation of breastfeeding, was assessed based on the time when the newborn was placed on the mother’s chest immediately after birth for skin-to-skin contact (2). If the respondent answered 1 h or more, the infant was categorized as having a delayed initiation of breastfeeding. The second outcome, prelacteal feeding, refers to the practice of giving infants food/liquid before breastfeeding is initiated (within 3 days of birth) (22). The third outcome, non-exclusive breastfeeding, was defined as infants who reported having consumed any food/liquid other than breastmilk (not including oral rehydration solution, drops, or syrups) in the last 24 h preceding the survey (2).

### 2.3. Potential predictors

**Figure 1:**
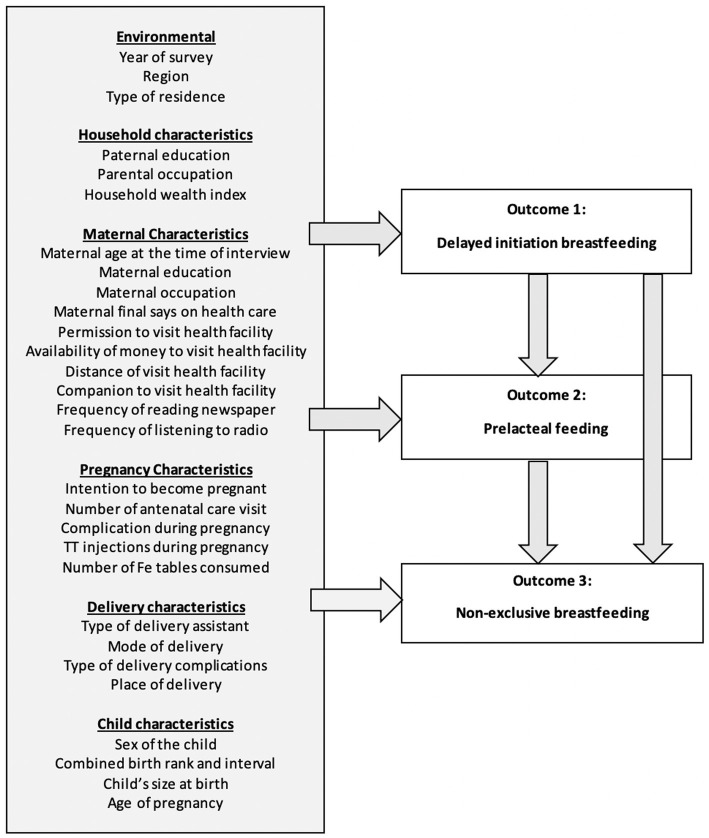
Potential predictors analyzed for factors associated with suboptimal early feeding practices in infants aged 0–5 months in Indonesia.

Our analysis used 29 potential predictors of delayed initiation of breastfeeding. These variables were classified into six groups: (1) environmental, (2) household, (3) maternal, (4) pregnancy, (5) delivery, and (6) child characteristics. For prelacteal feeding, we included an additional variable of delayed initiation of breastfeeding. For non-exclusive breastfeeding, two additional variables were included, i.e., delayed initiation of breastfeeding and prelacteal feeding (Figure 1).

Using the principal component analysis method (23), we constructed a household wealth index using 11 housing variables and household assets: primary material of the floor and wall, type of toilet, availability of electricity, source of drinking water, and possession of radio, television, fridge, bicycle, motorcycle, and car. This was followed by constructing a five-category household wealth index variable: the poorest, poor, middle, rich, and richest.

The variable of ‘maternal final says on health care’ was constructed based on respondents’ responses about the person who usually decides on the respondent’s health care. The answers were grouped into women alone, women with her partner, and partners alone. The variable of ‘child size at birth’ was used to reflect the maternal subjective assessment of the infant’s size, due to the high missing value of birth weight at birth in the IDHS.

### 2.4. Statistical analysis

Initially, the characteristics of all infants included in this analysis were examined using frequency tabulations. The univariate logistic regression analysis was performed without adjusting for other covariates to assess the crude association between each potential predictor and study outcome.

The multivariable regression analysis was conducted to investigate the relationship between potential predictors and study outcomes by controlling for other covariates. The backward elimination procedure was employed to select factors significantly associated with each study outcome (significance level of 0.05). All potential predictors were included in the baseline model. We retained all variables significantly associated with study outcomes in the final model in addition to some variables selected *a priori* regardless of their significance level, i.e., the year of IDHS, region, and type of residence (urban/rural). This study calculated odds ratios (ORs) and 95% confidence intervals (CIs). All estimates were weighted by sampling probabilities. STATA/MP version 16.0 was used for all statistical analyses (Stata Corporation, College Station, TX, United States).

This is a secondary analysis of the IDHS data available in the public domain; therefore, ethical approval is not required. The study was conducted according to internationally agreed ethical principles for medical research.

## 3. Results

**Figure 2:**
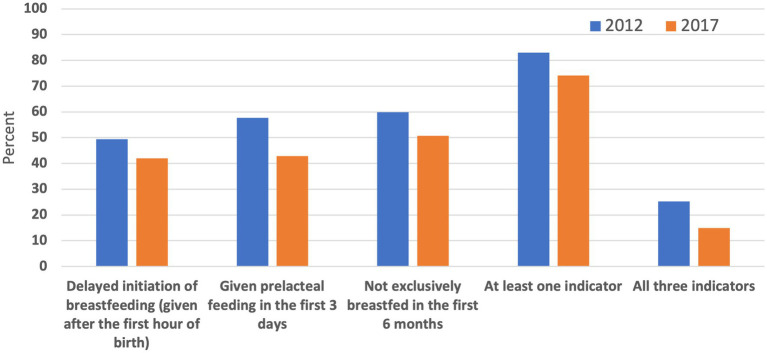
The changes in the prevalence of suboptimal early feeding practices in infants aged 0–5 months in Indonesia, The 2012 and 2017 IDHS.

Of the 3,198 infants aged 0–5 months analyzed in this study, approximately 78.6% (95% CI: 76.65–80.8) had at least one of the three suboptimal early infant feeding practices evaluated in this study. Approximately 45.77% (95% CI: 43.44–48.11) infants were not put to their mother’s breast in the first hour of life; 50.43% (95% CI: 47.98–52.87) infants received prelacteal feeding in the first 3 days of life, and 55.32% (95% CI: 52.99–57.63) were not exclusively breastfed in the last 24 h preceding the survey. There were 20.15% (95% CI: 18.37–22.05) of infants who had all three suboptimal feeding practices. Figure 2 shows the changes in the three outcome indicators between the 2012 and the 2017 IDHS.

The frequency distribution of variables used in this analysis and their distribution by each outcome variable is presented in Table 1. Around 57% of the mothers did not work outside the house, 86% had at least four antenatal visits and 90% were assisted by trained attendants at delivery. Supplementary Table S1 shows the results of the univariable analysis of factors associated with each study outcome.

**Table 1:** Frequency distribution of variables analyzed by three suboptimal early infant feeding practices (delayed initiation of breastfeeding, prelacteal feeding, and non-exclusive breastfeeding in the last 24 h preceding the survey), The 2012 and 2017 IDHS.

Variable	Frequency		Prevalence		
			Delayed initiation of breastfeeding	Prelacteal Feeding	Non-exclusive breastfeeding in the last 24 h
	*n*	%	%	%	%
Suboptimal infant feeding practice					
**Non-exclusive breastfeeding**					
No	1428	44.68	35.79	36.44	
Yes	1769	55.32	64.21	63.56	
**Prelacteal feeding**					
No	1585	49.57	30.95		46.94
Yes	1612	50.43	69.05		63.56
**Delayed initiation of breastfeeding**					
No	1463	45.77		69.05	35.79
Yes	1734	54.23		34.71	52.17
Environmental characteristics					
**Year of survey**					
2012	1621	50.68	49.42	57.76	59.82
2017	1577	49.32	42.01	42.89	50.7
**Region**					
Java-Bali	1711	53.52	41.41	48.33	46.59
Sumatera	759	23.75	57.88	60.03	38.86
Eastern Indonesia	727	22.73	43.37	45.32	46.26
**Type of place of residence**					
Rural	1532	47.93	46.55	49.19	41.45
Urban	1665	52.07	45.04	51.56	47.64
Household-level characteristics					
**Husband’s education**					
No education	37	1.15	57.15	42.62	33.02
Incomplete primary school	205	6.41	39.56	43.01	48.51
Completed primary school	672	21.01	44.95	48.29	45.35
Incomplete secondary school	753	23.54	45.11	47.98	47.29
Secondary school or above	1482	46.36	47.06	54.42	43.35
**Husband’s occupation**					
Non agriculture	2416	75.56	45.83	51.08	43.69
Agriculture	700	21.90	45.97	50.97	48.98
Not working	28	0.88	47.84	25.57	43.52
**Household wealth index**					
Poorest	532	16.63	40.5	43.39	49.67
Poorer	642	20.08	46.11	46.8	45.6
Middle	791	24.73	46.66	49.44	44.34
Richer	592	18.51	45.93	54.27	40.13
Richest	485	15.17	47.13	58.91	44.85
Maternal characteristics					
**Maternal age at the time of interview**					
Less than 20 years old	219	6.86	49.09	56.58	41.2
20–29 years old	1646	51.47	46.32	51.37	44.95
30–39 years old	1169	36.57	44.01	48.5	44.98
40 or more years old	163	5.10	48.25	46.5	44.45
**Maternal education**					
No education	55	1.71	32.85	27.83	26.36
Incomplete primary school	199	6.23	44.84	41.39	45.82
Completed primary school	609	19.06	42.81	50.41	46.15
Incomplete secondary school	898	28.09	46.86	48.38	44.49
Secondary school or above	1436	44.91	46.96	53.83	44.71
**Maternal occupation**					
Not working	1831	57.27	44.17	49.53	48.68
Agriculture	210	6.57	43.71	40.43	46.22
Non agriculture	1156	36.16	48.67	53.66	38.05
**Maternal final says on health care**					
Woman alone	1139	35.63	44.71	49.67	44.32
Woman with partner	1553	48.59	46.21	52.06	45.62
Partner alone	456	14.26	47.74	50.49	43.45
**Permission to visit health care facility**					
Not concerned	2996	93.70	45.44	50.57	44.53
Concerned	194	6.07	51.08	48.86	45.97
**Availability of money to visit health care facility**					
Not concerned	2719	85.03	45.51	51.35	44.82
Concerned	470	14.69	47.24	45.28	43.59
**Distance to visit health care facility**					
Not concerned	2822	88.26	45.37	50.63	44.78
Concerned	366	11.43	48.93	49.28	43.43
**Companion to visit health care facility**					
Not concerned	2381	74.45	44.56	50.72	45.06
Concerned	808	25.26	49.29	49.65	43.42
**Frequency of reading newspaper**					
At least once a week	307	9.61	45.82	51.35	35.21
Less than once a week	1134	35.47	48.14	54.11	45.02
Never	1748	54.68	44.21	47.88	46.15
**Frequency of listening to radio**					
At least once a week		12.57	45.58	53.38	40.17
Less than once a week		32.92	46.52	56.5	43.19
Never		54.49	45.34	46.09	46.6
Pregnancy characteristics					
**Intention to become pregnant**					
Then	2599	81.29	45.47	51.43	43.71
Later	324	10.12	45.6	44.43	53.91
No more	268	8.37	47.52	49.28	43.95
**Antenatal care visit**					
Four or more visits	2753	86.11	44.74	51.21	46.27
Less than four visits	444	13.89	52.15	45.55	34.81
**Complications during pregnancy**					
Without complication	2684	83.95	45.71	50.11	45.25
With complication	513	16.05	46.08	52.08	41.67
**Tetanus Toxoid injections during pregnancy**					
Never	1090	34.10	49.7	53.19	41.79
One injection	877	27.43	43.1	49.18	49.69
Two or more injections	1180	36.91	43.71	49.35	43.98
Do not know	40	1.26	47.2	45.96	44.35
**Number of iron tablets consumed**					
None	585	18.30	46.15	47.28	39.23
<90	1219	38.11	46.85	51.67	43.39
90–179	562	17.57	40.04	49.85	49.52
180+	683	21.35	45.27	51.91	51.46
Do not know	105	3.27	57.85	52.53	30.31
Delivery characteristics					
**Delivery assistant**					
Health professional	2893	90.49	45.76	50.9	44.88
None/traditional birth attendants	288	9.00	45.85	47.54	43.59
**Mode of delivery**					
Vaginal delivery	2651	82.93	41.99	47.78	46.64
Cesarean Section	546	17.07	64.1	63.3	35.14
**Type of delivery complications**					
None	1231	38.50	43.5	51.72	43.73
Any complication	1804	56.43	47.2	49.58	45.98
**Place of delivery**					
Public health facilities	1176	34.27	43.62	46.09	52.72
Private health facilities	1279	37.27	48.94	55.12	59.97
Non-health facilities (e.g., at home or other places outside the health facility)	977	28.47	48.62	50.46	59.16
Child characteristics					
**Sex of the child**					
Female	1579	49.39	45.31	49.93	46.17
Male	1618	50.61	46.21	50.91	43.22
**Combined birth rank and birth interval**					
2nd/3rd birth rank, more than 2-years interval	1445	45.20	42.2	48.06	48.06
1st birth rank	1176	36.78	52.53	57.54	38.93
2nd/3rd birth rank, less than or equal 2-years interval	160	5.00	36.36	44.65	56.6
4th birth rank, more than 2-years interval	338	10.57	45.27	42.45	43.61
4th birth rank, less than or equal to 2-years interval	79	2.46	31.43	33.64	48.81
**Child’s size at birth**					
Average	1787	55.88	44.77	50.65	44.35
Smaller than average	397	12.40	52.29	54.03	41.35
Larger than average	937	29.30	44.12	48.94	47.31
**Age of pregnancy**					
Term	3116	97.47	45.85	50.27	44.93
Preterm	76	2.38	42.31	59.66	36.85

Table 2 presents the results of the multivariable analysis of factors associated with each outcome indicator in this analysis. For the delayed initiation of breastfeeding, we found a significant reduction in the odds in **2017 IDHS** compared to 2012 IDHS (aOR = 0.77, 95% CI: 0.62–0.95), infants from **Sumatera Region** (aOR = 2.02, 95% CI: 1.59–2.57), and **from Eastern Indonesia** (aOR = 1.36, 95% CI: 1.07–1.73). In household and maternal level characteristics, infants whose fathers had a high **level of education** had reduced odds. In contrast, infants whose mothers had a high level of education had increased odds of delayed initiation of breastfeeding. Our study also showed increased odds in infants who had less than **four antenatal visits** (aOR = 1.56, 95% CI: 1.16–2.11), delivered by **Cesarean Section** (aOR = 2.78, 95% CI: 2.07–3.73), and **delivered in non-health facilities** (aOR = 1.53, 95% CI: 1.15–2.03). Increased odds were also associated with the **first birth rank infants** (aOR = 1.56, 95% CI: 1.24–1.97).

**Table 2:** Factors associated with suboptimal early feeding practices in infants aged 0–5 months, The 2012 and 2017 IDHS.

Variable	Delayed initiation of breastfeeding				Prelacteal feeding				Non-exclusive breastfeeding in the last 24 h			
	aOR	95% CI		*p*	aOR	95% CI		*p*	aOR	95% CI		*p*
Suboptimal infant feeding practice												
**Prelacteal feeding**												
No									1.00			
Yes									1.62	1.30	2.00	*<0.001*
**Delayed initiation of breastfeeding**												
No					1.00				1.00			
Yes					3.90	3.17	4.79	*<0.001*	1.47	1.18	1.82	*<0.001*
Environmental characteristics												
**Year of survey**												
2012	1.00				1.00				1.00			
2017	0.77	0.62	0.95	*0.014*	0.57	0.46	0.72	*<0.001*	0.78	0.63	0.96	*0.018*
**Region**												
Java-Bali	1.00				1.00				1.00			
Sumatera	2.02	1.59	2.57	*<0.001*	1.45	1.10	1.89	*0.007*	1.29	1.02	1.63	*0.033*
Eastern Indonesia	1.36	1.07	1.73	*0.013*	1.00	0.77	1.29	*0.975*	1.15	0.91	1.45	*0.231*
**Type of place of residence**												
Rural	1.00				1.00				1.00			
Urban	0.95	0.74	1.21	*0.673*	1.32	1.02	1.70	*0.035*	1.36	1.08	1.70	*0.008*
Household-level characteristics												
**Husband’s education**												
No education	1.00								1.00			
Incomplete primary school	0.31	0.13	0.73	*0.007*					0.39	0.19	0.79	*0.009*
Completed primary school	0.44	0.19	1.01	*0.053*					0.39	0.20	0.77	*0.007*
Incomplete secondary school	0.37	0.16	0.85	*0.020*					0.36	0.18	0.72	*0.004*
Secondary school or above	0.38	0.17	0.88	*0.024*					0.32	0.16	0.63	*0.001*
**Husband’s occupation**												
Non agriculture					1.00							
Agriculture					1.03	0.78	1.36	*0.838*				
Not working					0.26	0.11	0.60	*0.002*				
**Household wealth index**												
Poorest	1.00				1.00				1.00			
Poorer	1.29	0.95	1.76	*0.104*	1.05	0.75	1.47	*0.775*	1.27	0.93	1.73	*0.136*
Middle	1.38	1.00	1.90	*0.050*	1.26	0.90	1.75	*0.172*	1.32	0.96	1.80	*0.085*
Richer	1.37	0.95	1.98	*0.092*	1.63	1.13	2.36	*0.009*	1.60	1.14	2.24	*0.007*
Richest	1.41	0.92	2.14	*0.112*	2.05	1.34	3.15	*0.001*	1.21	0.83	1.75	*0.322*
Maternal characteristics												
**Maternal education**												
No education	1.00											
Incomplete primary school	2.77	1.14	6.73	*0.025*								
Completed primary school	2.53	1.13	5.70	*0.025*								
Incomplete secondary school	2.87	1.27	6.48	*0.011*								
Secondary school or above	2.60	1.13	6.00	*0.025*								
**Maternal occupation**												
Not working									1.00			
Agriculture									1.02	0.69	1.51	*0.935*
Non agriculture									1.52	1.21	1.90	*<0.001*
Pregnancy characteristics												
**Antenatal care visit**												
Four or more visits	1.00								1.00			
Less than four visits	1.56	1.16	2.11	*<0.001*					1.80	1.36	2.38	*<0.001*
Delivery characteristics												
**Mode of Delivery**												
Vaginal delivery	1.00								1.00			
Cesarean Section	2.78	2.07	3.73	*<0.001*					1.33	1.01	1.75	*0.044*
**Place of delivery**												
Public health facilities	1.00											
Private health facilities	1.24	0.96	1.60	*0.098*								
Non-health facilities (e.g., at home or other places outside the health facility)	1.53	1.15	2.03	*0.003*								
Child characteristics												
**Combined birth rank and birth interval**												
2nd/3rd birth rank, more than 2-years interval	1.00				1.00				1.00			
1st birth rank	1.56	1.24	1.97	*<0.001*	1.32	1.05	1.66	*0.019*	1.28	1.01	1.62	*0.039*
2nd/3rd birth rank, less than or equal 2-years interval	0.68	0.44	1.07	*0.093*	0.86	0.53	1.40	*0.542*	0.60	0.38	0.95	*0.030*
4th birth rank, more than 2-years interval	1.01	0.72	1.40	*0.976*	0.72	0.52	1.01	*0.058*	1.11	0.82	1.50	*0.494*
4th birth rank, less than or equal to 2-years interval	0.54	0.28	1.03	*0.060*	0.58	0.28	1.21	*0.148*	0.86	0.46	1.61	*0.642*

Only variables significantly associated with study outcomes were retained in the final model.

Year of IDHS, region, and type of residence (urban/rural) were selected a priori to be retained in the final model regardless of their significance level.

For the prelacteal feeding practice, similar to the delayed initiation of breastfeeding practice, we found significantly lower odds in **2017 than** in 2012 IDHS (aOR = 0.57, 95%CI: 0.46–0.72) (Table 2). Amongst the environmental-level characteristics, increased odds of prelacteal feeding was found in infants living in **Sumatera Region** (aOR = 1.45, 95% CI: 1.10–1.89) and in **urban areas** (aOR = 1.32, 95% CI: 1.02–1.70). Amongst the household-level characteristics, a reduced odds of delayed initiation of breastfeeding was found in infants of **not-working fathers** (aOR = 0.26, 95% CI: 0.11–0.60), while an increased odds was associated with infants born to mothers from **wealthy households**. An increased odds for prelacteal feeding was also associated with the **first birth rank infants** (aOR = 1.32, 95% CI: 1.05–1.66) and infants who reported having **delayed initiation of breastfeeding** in the first hour of life (aOR = 3.90, 95% CI: 3.17–4.79).

For the non-exclusive breastfeeding practice in the last 24 h preceding the survey, our multivariable analysis showed significantly lower odds in **2017** than in 2012 IDHS (aOR = 0.78, 95% CI: 0.63–0.96) (Table 2). Among the environmental and household-level characteristics, increased odds was found in infants living in **Sumatera Region** (aOR = 1.29, 95% CI: 1.02–1.63) and **urban areas** (aOR = 1.36, 95% CI: 1.08–1.70). We found reduced odds of non-exclusive breastfeeding amongst infants whose **fathers were educated**, while increased odds were found in mothers from **richer households**. Regarding maternal characteristics, the odds of non-exclusive breastfeeding increased significantly in **mothers working in the non-agricultural field** (aOR = 1.52, 95% CI: 1.21–1.90). Regarding the pregnancy and delivery characteristics, increased odds were associated with mothers attending **less than four antenatal care services** (aOR = 1.80, 95% CI: 1.36–2.38), mothers **delivered by Cesarean section** (aOR = 1.33, 95% CI: 1.01–1.75), and among the **first birth-ranked infants** (aOR = 1.28, 95% CI: 1.01–1.62). An increased odds for non-exclusive breastfeeding in the last 24 h was also associated with infants who had **delayed initiation of breastfeeding** (aOR = 1.47, 95% CI: 1.18–1.82) in the first hour of life and with those who received **prelacteal feeding** in the first 3 days of life (aOR = 1.62, 95% CI: 1.30–2.00).

## 4. Discussion

### 4.1. Main findings

Our study showed that approximately three-quarters of the infants aged 0–5 months in Indonesia had at least one of the suboptimal early infant feeding practices examined in this analysis (either delayed initiation of breastfeeding in the first hour of life, prelacteal feeding in the first 3 days of life, or not exclusively breastfed in the past 24 h preceding the survey). The determinants of delayed initiation of breastfeeding in the first hour of life were the year of IDHS, the region where infants lived, the father’s and mother’s level of education, antenatal visit, mode of delivery, place of delivery, and birth rank. Our study also found different factors associated with prelacteal breastfeeding practices in the first 3 days of life, i.e., the year of IDHS, the region where infants lived, type of residence, fathers’ occupation, household wealth index, birth rank, and timely initiation of breastfeeding. The predictors of exclusive breastfeeding practice in the last 24 days preceding the survey found in our analysis were the year of IDHS, the region where infants lived, type of residence, fathers’ education, household wealth index, mother’s occupation, antenatal visits, mode of delivery, birth rank, timely initiation of breastfeeding and prelacteal feeding practices. Our study showed the association between different factors and suboptimal early infant feeding practices. This could be used by program managers to formulate evidence-based interventions, including programmatic actions to overcome barriers to adopting recommended early infant feeding practices in Indonesia.

### 4.2. Increasing knowledge and awareness of optimal feeding practice

Our analysis found an increased likelihood for suboptimal early infant feeding practices in infants from Sumatera and the Eastern part of Indonesia, compared to those from the Java-Bali region. Lower access to health care services or health information in Sumatera and the Eastern part of Indonesia could contribute to the suboptimal early infant feeding practices. The disparity in access to and use of health services in those areas was also reported in previous studies (24, 25). Interestingly, although access to services and information is more likely to be limited in rural areas, our findings showed that infants from urban areas were more likely to be fed sub-optimally. An earlier study reported a similar finding showing better feeding practices among infants in rural than urban areas (26). The availability and accessibility of various breastmilk substitutes, heavily marketed in urban areas, could also explain this finding. Additionally, high female participation in the workforce in urban areas (27) may force mothers in cities to feed their infants with breastmilk substitutes.

Furthermore, this analysis found an association between the household wealth index and early infant feeding practices. An increased likelihood of infants having suboptimal early infant feeding practices when their mothers were from a high household wealth index could reflect the household’s economic capability to provide prelacteal food and formula milk as breastmilk substitutes. Additionally, as reported in previous literature (28, 29), we found that infants born to mothers with a high educational level were more likely to have suboptimal feeding practices. Mothers with a high education level will be more likely to have a high household wealth index and thus, could afford formula milk. This could further be aggravated by the fact that in some communities, formula milk is considered a marker of prestige (30).

The first birth-rank infants in this study were also more likely to have delayed initiation of breastfeeding, receive prelacteal feeding, and not be exclusively breastfed. This could be attributable to the difference in the knowledge between multiparous and primiparous women. Multiparous mothers were considered more knowledgeable and experienced in breastfeeding than primiparous women (31).

All these findings demonstrated the need for effective health promotion strategies in raising community awareness about the critical role of appropriate infant and young child feeding practices, including during early infancy. The educational intervention should specifically target mothers living in the Sumatera and Eastern part of Indonesia region, from rural areas, with a high household wealth index, high education level, as well as primiparous mothers. Previous literature showed that there were various effective educational interventions that could promote mothers’ self-efficacy and optimal breastfeeding practices, including those based on self-efficacy and planned behavior theory or internet technologies (32, 33).

The significant association between the three early infant feeding practices analyzed in this study also emphasizes the need to target women early, for example, during antenatal care services. This was = reflected in this analysis as women who attended fewer than four antenatal visits were more likely to delay breastfeeding initiation and not exclusively breastfeed their infants. Various studies also showed a significant association between antenatal care services and optimal breastfeeding practices (34). Mothers with adequate antenatal visits had increased opportunities to receive various health-related education, including lactation guidance and counseling. According to a study conducted in Lao PDR, around half of the mothers intended to purchase infant formula after watching its television advertisements (35). This poses a great challenge to health workers to consistently educate the community to make the most appropriate feeding for infants. Therefore, health workers should possess adequate knowledge and counseling skills to educate mothers and their family members. Health workers should also take advantage of every contact opportunity to promote optimal infant and young child feeding practices.

### 4.3. Supportive public policy

As breastfeeding requires collective social responsibilities to remove barriers to its practice, our findings highlight the need for a supportive public policy to encourage mothers to have optimal infant feeding practices. Our analysis shows that working mothers were more likely than non-working mothers not exclusively to breastfeed their infants. This was consistent with our previous study using the 2002/2003 and 2017 IDHS (16).

The improved optimal breastfeeding practices between 2012 and 2017 IDHS could reflect some of the supportive policies and regulations the Government of Indonesia has introduced since 2003 (36). This includes the 2003 Labor Law that forces employers to provide 3 months of paid maternity leave and allow breastfeeding breaks for working women. Later in 2009, a Joint Regulation from the Ministry of Women’s Empowerment, the Ministry of Manpower and Transmigration, and the Ministry of Health were issued that highlighted the benefits of breastfeeding and endorsed the provision of lactation rooms as well as breastfeeding breaks at workplaces during working hours (37). Furthermore, the government targeted the minimum rate of 50% for early initiation of breastfeeding and exclusive breastfeeding in the Strategic Plan of the Ministry of Health 2015–2019 (38).

However, despite the improvement, there are still challenges encountered in implementing these policies. For example, the maternity leave policy only regulates 13 weeks of paid maternity leave (39). This limits the ability of mothers to exclusively breastfeed their infants for 6 months. It was further reported that this regulation was not yet optimally enforced, as many eligible women still did not receive it (40). At workplaces, unclear policies at the workplace were also reported, for example, whether mothers could use their rest breaks at work to express breastmilk or the unavailability of lactation rooms at work (41). If the guidelines existed, the implementation was sometimes unclear and ineffective (42). Moreover, it is also important to formulate policies protecting women working in informal sectors, which remain unregulated.

One of the most complicated challenges for optimal breastfeeding practice is the aggressive and inappropriate marketing of breastmilk substitutes. Despite the World Health Assembly’s (WHA) adoption of the International Code of Marketing of Breastmilk Substitutes (The Code) in 1981, which outlines the international policy framework for protecting breastfeeding from unethical marketing practices (43), many countries continue to face difficulties in incorporating The Code into their legislation (44). A study in Indonesia examining compliance with The Code found violations among breastmilk substitute companies as well as health workers in all studied sites (45). Approximately 72% of women saw breastmilk substitute promotional materials in healthcare facilities. This points to the need for a monitoring system to ensure improved Code compliance and enforcement, as well as to raise awareness among stakeholders, including the general community, health workers, government, and manufacturers.

### 4.4. Creating supportive environments

An increased likelihood of delayed initiation of breastfeeding or non-exclusive breastfeeding in women who delivered through Cesarean section, as reported in other studies, was confirmed in our analysis (46, 47). This could be related to the problem of lactogenesis due to abdominal surgery. The Cesarean section might disrupt the hormonal pathway that spurs lactogenesis because of maternal stress or lowers oxytocin secretion (48). Delivery complications that were more likely to occur in the first pregnancy could force women to have a Cesarean section. Consequently, mothers could be separated from their infants, leading to delayed breastfeeding initiation in the first hour after delivery (31). Some studies reported that routines care after a Cesarean section interfered with bonding and delayed mothers holding their infants (49). Furthermore, some hospital settings lacked a specific breastfeeding protocol and were not Baby Friendly Initiatives certified (50). This shows that supportive care should begin immediately after delivery and continue until the early postpartum period for mothers with Cesarean sections. The availability and access to trained lactation consultants and other breastfeeding support are critical to promoting early initiation of breastfeeding and long-term breastfeeding success.

Furthermore, we found an increased likelihood of delayed breastfeeding initiation among those delivered in non-health facilities. In Indonesia, although more than 20% of deliveries in Indonesia still occurred at home, more than 90% of deliveries were already attended by skilled birth attendants (15). This shows the need for skilled birth attendants to continually promote optimal breastfeeding practices, including for mothers delivered outside health facilities.

### 4.5. Strengths and limitations of the study

The study had several strengths. First, this study used two nationally representative survey datasets with a large sample size to investigate the role of various predictors of early feeding practices in infants aged 0–5 months. Second, the IDHS also used a standardized questionnaire and methods that could be used to compare results across years of surveys and countries. However, when interpreting the findings of this study, several limitations should be considered. We used 24 h recall data for exclusive breastfeeding; therefore, the rate of exclusive breastfeeding might be overestimated. Some recall bias might occur, as mothers of older infants might forget details in their infants’ early days of life. However, our analysis used information only from mothers of infants aged 0–5 months, which could minimize the bias. The information provided in this study was not validated as in other cross-sectional surveys. Nevertheless, these limitations did not affect the validity of our results.

### 4.6. Conclusion

Our study found a high percentage of infants aged 0–5 months who had suboptimal feeding practices in Indonesia. There was a significant association between environmental, household, maternal, pregnancy, delivery, and child characteristics with delayed initiation of breastfeeding in the first hour of life, prelacteal feeding in the first 3 days of life, and non-exclusive breastfeeding in the last 24 h preceding the survey. This highlights the importance of an integrated approach to ensure that infants receive adequate nutrition for their health, growth, and development in their early years. Effective health education interventions, using different channels of communication as well as through antenatal care services, are critical in raising mothers, other family members, and the general community’s awareness and understanding of the importance of optimal early infant feeding practices. Consequently, improving health workers’ awareness, knowledge, and skills is critical to conduct effective counseling and other health promotion strategies. Moreover, efforts to create supportive public policies, such as maternal leave regulations or the implementation of the International Code of Marketing of Breastmilk Substitutes, should be accompanied by continuous monitoring and rigorous enforcement. Furthermore, breastfeeding protocol and Baby Friendly Hospital Initiatives could be continuously promoted at health facilities to better support breastfeeding practices in Indonesia.

## Data availability statement

The datasets presented in this study can be found in online repositories. The names of the repository/repositories and accession number (s) can be found below: The DHS Program (https://dhsprogram.com/data/available-datasets.cfm).

## Ethics statement

Ethical approval was not provided for this study on human participants because this is a secondary analysis of the Indonesia Demographic and Health Survey (IDHS) data available in the public domain; therefore, ethical approval is not required. The study was conducted according to internationally agreed ethical principles for medical research. The patients/participants provided their written informed consent to participate in this study.

## Author contributions

CT and RW conceived the study. CT, AM, and IA conducted data analysis. CT drafted the manuscript. RW and IA contributed to the literature review and provided advice on the data analysis. All authors contributed to the article and approved the submitted version.

## Conflict of interest

The authors declare that the research was conducted in the absence of any commercial or financial relationships that could be construed as a potential conflict of interest.

## Publisher’s note

All claims expressed in this article are solely those of the authors and do not necessarily represent those of their affiliated organizations, or those of the publisher, the editors and the reviewers. Any product that may be evaluated in this article, or claim that may be made by its manufacturer, is not guaranteed or endorsed by the publisher.
