# Supplementary material for: The multiple factors of suboptimal early feeding practices among infants aged 0–5 months in Indonesia
Source: Front Nutr. 2023 Mar 28;10:1080727. doi: 10.3389/fnut.2023.1080727 (PMC10086344; doi:10.3389/fnut.2023.1080727)
Supplement: Supplementary file 1 [file Table_1.docx]

**Supplementary Table 1**.

Results of the univariable logistic regression of factors associated with delayed initiation of breastfeeding, prelacteal feeding, and non-exclusive breastfeeding in the last 24 hours preceding the survey, The 2012 and 2017 IDHS

| **Variable** | **Delayed initiation of breastfeeding** | | | | **Prelacteal Feeding** | | | | **Non-exclusive breastfeeding**  **in the last 24 hours** | | | |
| --- | --- | --- | --- | --- | --- | --- | --- | --- | --- | --- | --- | --- |
|  | **Bivariate analysis** | | | | **Bivariate analysis** | | | | **Bivariate analysis** | | | |
|  | **OR** | **95% CI** | | ***p*** | **OR** | **95% CI** | | ***p*** | **OR** | **95% CI** | | ***p*** |
| **Suboptimal infant feeding practice** |  |  |  |  |  |  |  |  |  |  |  |  |
| **Non-exclusive breastfeeding** |  |  |  |  |  |  |  |  |  |  |  |  |
| No | *Variable not used in the model* | | | | *Variable not used in the model* | | | |  | | | |
| Yes |  |  |  |  |  |  |  |  |  |  |  |  |
| **Prelacteal Feeding** |  |  |  |  |  |  |  |  |  |  |  |  |
| No | *Variable not used in the model* | | | |  | | | | 1,00 |  |  |  |
| Yes |  |  |  |  |  |  |  |  | 1,97 | 1,63 | 2,38 | *<0,001* |
| **Delayed initiation of breastfeeding** |  |  |  |  |  |  |  |  |  |  |  |  |
| No |  | | | | 1,00 |  |  |  | 1,00 |  |  |  |
| Yes |  |  |  |  | 0,24 | 0,20 | 0,29 | *<0,001* | 1,96 | 1,62 | 2,36 | *<0,001* |
| **Environmental Characteristics** |  |  |  |  |  |  |  |  |  |  |  |  |
| **Year of survey** |  |  |  |  |  |  |  |  |  |  |  |  |
| 2012 | 1,00 |  |  |  |  |  |  |  | 1,00 |  |  |  |
| 2017 | 0,74 | 0,61 | 0,90 | *0,002* | 0,55 | 0,45 | 0,67 | *<0,001* | 0,69 | 0,57 | 0,84 | *<0,001* |
| **Region** |  |  |  |  |  |  |  |  |  |  |  |  |
| Java-Bali | 1,00 |  |  |  | 1,00 |  |  |  | 1,00 |  |  |  |
| Sumatera | 1,94 | 1,56 | 2,42 | *<0,001* | 1,61 | 1,28 | 2,01 | *<0,001* | 1,37 | 1,11 | 1,70 | *0,003* |
| Eastern Indonesia | 1,08 | 0,89 | 1,32 | *0,432* | 0,89 | 0,72 | 1,08 | *0,239* | 1,01 | 0,83 | 1,24 | *0,896* |
| **Type of place of residence** |  |  |  |  |  |  |  |  |  |  |  |  |
| Rural |  |  |  |  | 1,00 |  |  |  | 1,00 |  |  |  |
| Urban | 1,06 | 0,87 | 1,30 | *0,548* | 0,91 | 0,75 | 1,11 | *0,348* | 1,29 | 1,06 | 1,55 | *0,010* |
| **Household-level Characteristics** |  |  |  |  |  |  |  |  |  |  |  |  |
| **Husband's education** |  |  |  |  |  |  |  |  |  |  |  |  |
| No education | 1,00 |  |  |  | 1,00 |  |  |  | 1,00 |  |  |  |
| Incomplete primary school | 0,49 | 0,22 | 1,07 | *0,075* | 1,02 | 0,47 | 2,22 | *0,968* | 0,52 | 0,23 | 1,18 | *0,117* |
| Completed primary school | 0,61 | 0,30 | 1,27 | *0,187* | 1,26 | 0,61 | 2,58 | *0,533* | 0,59 | 0,27 | 1,29 | *0,186* |
| Incomplete secondary school | 0,62 | 0,30 | 1,27 | *0,189* | 1,24 | 0,61 | 2,53 | *0,550* | 0,55 | 0,25 | 1,19 | *0,130* |
| Secondary school or above | 0,67 | 0,33 | 1,36 | *0,267* | 1,61 | 0,79 | 3,25 | *0,187* | 0,64 | 0,30 | 1,38 | *0,258* |
| **Husband's occupation** |  |  |  |  |  |  |  |  |  |  |  |  |
| Non agriculture | 1,00 |  |  |  | 1,00 |  |  |  | 1,00 |  |  |  |
| Agriculture | 1,01 | 0,81 | 1,25 | *0,961* | 1,00 | 0,80 | 1,25 | *0,971* | 0,81 | 0,65 | 1,01 | *0,059* |
| Not working | 1,08 | 0,48 | 2,45 | *0,846* | 0,33 | 0,16 | 0,68 | *0,003* | 1,01 | 0,42 | 2,44 | *0,988* |
| **Household Wealth Index** |  |  |  |  |  |  |  |  |  |  |  |  |
| Poorest | 1,00 |  |  |  | 1,00 |  |  |  | 1,00 |  |  |  |
| Poorer | 1,26 | 0,95 | 1,67 | *0,112* | 1,15 | 0,85 | 1,54 | *0,360* | 1,18 | 0,89 | 1,57 | *0,261* |
| Middle | 1,28 | 0,98 | 1,68 | *0,070* | 1,28 | 0,96 | 1,70 | *0,094* | 1,24 | 0,94 | 1,63 | *0,128* |
| Richer | 1,25 | 0,92 | 1,69 | *0,152* | 1,55 | 1,14 | 2,11 | *0,005* | 1,47 | 1,09 | 1,98 | *0,011* |
| Richest | 1,31 | 0,94 | 1,82 | *0,109* | 1,87 | 1,33 | 2,64 | *<0,001* | 1,21 | 0,88 | 1,67 | *0,238* |
| **Maternal Characteristics** |  |  |  |  |  |  |  |  |  |  |  |  |
| **Maternal age at the time of interview** |  |  |  |  |  |  |  |  |  |  |  |  |
| Less than 20 years old | 1,00 |  |  |  | 1,00 |  |  |  | 1,00 |  |  |  |
| 20-29 years old | 0,90 | 0,62 | 1,29 | *0,549* | 0,81 | 0,56 | 1,17 | *0,259* | 0,86 | 0,57 | 1,29 | *0,460* |
| 30-39 years old | 0,82 | 0,56 | 1,20 | *0,296* | 0,72 | 0,50 | 1,04 | *0,082* | 0,86 | 0,57 | 1,28 | *0,451* |
| 40 or more years old | 0,97 | 0,55 | 1,69 | *0,906* | 0,67 | 0,39 | 1,13 | *0,130* | 0,88 | 0,49 | 1,56 | *0,653* |
| **Maternal education** |  |  |  |  |  |  |  |  |  |  |  |  |
| No education | 1,00 |  |  |  | 1,00 |  |  |  | 1,00 |  |  |  |
| Incomplete primary school | 1,66 | 0,80 | 3,45 | *0,173* | 1,83 | 0,87 | 3,87 | *0,113* | 0,42 | 0,22 | 0,83 | *0,012* |
| Completed primary school | 1,53 | 0,79 | 2,97 | *0,210* | 2,64 | 1,35 | 5,17 | *0,005* | 0,42 | 0,22 | 0,79 | *0,007* |
| Incomplete secondary school | 1,80 | 0,94 | 3,47 | *0,078* | 2,43 | 1,26 | 4,68 | *0,008* | 0,45 | 0,24 | 0,83 | *0,011* |
| Secondary school or above | 1,81 | 0,95 | 3,46 | *0,072* | 3,02 | 1,57 | 5,82 | *0,001* | 0,44 | 0,24 | 0,81 | *0,008* |
| **Maternal occupation** |  |  |  |  |  |  |  |  |  |  |  |  |
| Not working | 1,00 |  |  |  | 1,00 |  |  |  | 1,00 |  |  |  |
| Agriculture | 0,98 | 0,71 | 1,36 | *0,910* | 0,69 | 0,49 | 0,97 | *0,034* | 1,10 | 0,78 | 1,56 | *0,571* |
| Non agriculture | 1,20 | 0,98 | 1,46 | *0,078* | 1,18 | 0,96 | 1,44 | *0,107* | 1,54 | 1,27 | 1,88 | *<0,001* |
| **Maternal final says on health care** |  |  |  |  |  |  |  |  |  |  |  |  |
| Woman alone | 1,00 |  |  |  | 1,00 |  |  |  | 1,00 |  |  |  |
| Woman with partner | 1,06 | 0,87 | 1,30 | *0,562* | 1,10 | 0,90 | 1,35 | *0,356* | 0,95 | 0,77 | 1,17 | *0,628* |
| Partner alone | 1,13 | 0,85 | 1,50 | *0,395* | 1,03 | 0,78 | 1,37 | *0,817* | 1,04 | 0,76 | 1,41 | *0,820* |
| **Permission to visit health care facility** |  |  |  |  |  |  |  |  |  |  |  |  |
| Not concerned | 1,00 |  |  |  | 1,00 |  |  |  | 1,00 |  |  |  |
| Concerned | 1,25 | 0,89 | 1,76 | *0,190* | 0,93 | 0,66 | 1,32 | *0,700* | 0,94 | 0,65 | 1,36 | *0,756* |
| **Availability of money to visit health care facility** |  |  |  |  |  |  |  |  |  |  |  |  |
| Not concerned | 1,00 |  |  |  | 1,00 |  |  |  | 1,00 |  |  |  |
| Concerned | 1,07 | 0,84 | 1,36 | *0,571* | 0,78 | 0,61 | 1,01 | *0,059* | 1,05 | 0,82 | 1,34 | *0,689* |
| **Distance to visit health care facility** |  |  |  |  |  |  |  |  |  |  |  |  |
| Not concerned | 1,00 |  |  |  | 1,00 |  |  |  | 1,00 |  |  |  |
| Concerned | 1,15 | 0,89 | 1,49 | *0,275* | 0,95 | 0,73 | 1,22 | *0,680* | 1,06 | 0,80 | 1,39 | *0,696* |
| **Companion to visit health care facility** |  |  |  |  |  |  |  |  |  |  |  |  |
| Not concerned | 1,00 |  |  |  | 1,00 |  |  |  | 1,00 |  |  |  |
| Concerned | 1,21 | 0,99 | 1,48 | *0,065* | 0,96 | 0,78 | 1,18 | *0,686* | 1,07 | 0,86 | 1,33 | *0,552* |
| **Frequency of reading newspaper** |  |  |  |  |  |  |  |  |  |  |  |  |
| At least once a week | 1,00 |  |  |  | 1,00 |  |  |  | 1,00 |  |  |  |
| Less than once a week | 1,10 | 0,78 | 1,54 | *0,588* | 1,12 | 0,79 | 1,57 | *0,526* | 0,66 | 0,47 | 0,95 | *0,024* |
| Never | 0,94 | 0,67 | 1,31 | *0,702* | 0,87 | 0,62 | 1,22 | *0,416* | 0,63 | 0,46 | 0,88 | *0,007* |
| **Frequency of listening to radio** |  |  |  |  |  |  |  |  |  |  |  |  |
| At least once a week | 1,00 |  |  |  | 1,00 |  |  |  | 1,00 |  |  |  |
| Less than once a week | 1,04 | 0,76 | 1,43 | *0,815* | 1,13 | 0,83 | 1,54 | *0,420* | 0,88 | 0,64 | 1,23 | *0,457* |
| Never | 0,99 | 0,73 | 1,34 | *0,950* | 0,75 | 0,56 | 0,99 | *0,044* | 0,77 | 0,56 | 1,06 | *0,111* |
| **Pregnancy characteristics** |  |  |  |  |  |  |  |  |  |  |  |  |
| **Intention to become pregnant** |  |  |  |  |  |  |  |  |  |  |  |  |
| Then | 1,00 |  |  |  | 1,00 |  |  |  | 1,00 |  |  |  |
| Later | 1,01 | 0,74 | 1,37 | *0,973* | 0,75 | 0,56 | 1,02 | *0,065* | 0,66 | 0,49 | 0,90 | *0,010* |
| No more | 1,09 | 0,76 | 1,56 | *0,654* | 0,92 | 0,65 | 1,30 | *0,630* | 0,99 | 0,69 | 1,41 | *0,957* |
| **Antenatal care visit** |  |  |  |  |  |  |  |  |  |  |  |  |
| Four or more visits | 1,00 |  |  |  | 1,00 |  |  |  | 1,00 |  |  |  |
| Less than four visits | 1,35 | 1,05 | 1,73 | *0,020* | 0,80 | 0,62 | 1,02 | *0,068* | 1,61 | 1,25 | 2,07 | *<0,001* |
| **Complications during pregnancy** |  |  |  |  |  |  |  |  |  |  |  |  |
| Without complication | 1,00 |  |  |  |  |  |  |  | 1,00 |  |  |  |
| With complication | 1,02 | 0,79 | 1,30 | *0,904* | 1,08 | 0,85 | 1,38 | *0,523* | 1,16 | 0,91 | 1,47 | *0,226* |
| **Tetanus Toxoid injections during pregnancy** |  |  |  |  |  |  |  |  |  |  |  |  |
| Never | 1,00 |  |  |  |  |  |  |  | 1,00 |  |  |  |
| One injection | 0,77 | 0,61 | 0,97 | *0,028* | 0,85 | 0,67 | 1,08 | *0,183* | 0,73 | 0,57 | 0,93 | *0,012* |
| Two or more injections | 0,79 | 0,63 | 0,98 | *0,032* | 0,86 | 0,69 | 1,07 | *0,177* | 0,91 | 0,73 | 1,14 | *0,430* |
| Don't know | 0,90 | 0,40 | 2,03 | *0,808* | 0,75 | 0,33 | 1,68 | *0,481* | 0,90 | 0,39 | 2,10 | *0,809* |
| **Number of iron tablets consumed** |  |  |  |  |  |  |  |  |  |  |  |  |
| None | 1,00 |  |  |  |  |  |  |  | 1,00 |  |  |  |
| <90 | 1,03 | 0,80 | 1,32 | *0,825* | 1,19 | 0,94 | 1,51 | *0,150* | 0,84 | 0,64 | 1,11 | *0,218* |
| 90-179 | 0,78 | 0,57 | 1,07 | *0,119* | 1,11 | 0,82 | 1,50 | *0,508* | 0,66 | 0,48 | 0,90 | *0,009* |
| 180+ | 0,97 | 0,71 | 1,30 | *0,817* | 1,20 | 0,91 | 1,60 | *0,201* | 0,61 | 0,45 | 0,83 | *0,002* |
| Don't know | 1,60 | 0,93 | 2,76 | *0,089* | 1,23 | 0,73 | 2,09 | *0,434* | 1,48 | 0,84 | 2,61 | *0,171* |
| **Delivery characteristics** |  |  |  |  |  |  |  |  |  |  |  |  |
| **Delivery assistant** |  |  |  |  |  |  |  |  |  |  |  |  |
| Health professional | 1,00 |  |  |  |  |  |  |  | 1,00 |  |  |  |
| None/traditional birth attendants | 1,00 | 0,75 | 1,34 | *0,982* | 0,87 | 0,66 | 1,16 | *0,348* | 1,05 | 0,77 | 1,44 | *0,744* |
| **Mode of Delivery** |  |  |  |  |  |  |  |  |  |  |  |  |
| Vaginal delivery | 1,00 |  |  |  |  |  |  |  | 1,00 |  |  |  |
| Caesarean Section | 2,47 | 1,90 | 3,21 | *<0,001* | 1,89 | 1,46 | 2,44 | *<0,001* | 1,61 | 1,26 | 2,07 | *<0,001* |
| **Type of delivery complications** |  |  |  |  |  |  |  |  |  |  |  |  |
| None | 1,00 |  |  |  |  |  |  |  | 1,00 |  |  |  |
| Any complication | 1,16 | 0,96 | 1,41 | *0,132* | 0,92 | 0,75 | 1,13 | *0,421* | 0,91 | 0,75 | 1,11 | *0,365* |
| **Place of delivery** |  |  |  |  |  |  |  |  |  |  |  |  |
| Public health facilities | 1,00 |  |  |  | 1,00 |  |  |  | 1,00 |  |  |  |
| Private health facilities | 1,23 | 0,99 | 1,54 | *0,060* | 1,40 | 1,13 | 1,74 | *0,002* | 1,14 | 0,93 | 1,41 | *0,207* |
| Non-health facilities | 1,40 | 1,10 | 1,78 | *0,007* | 1,29 | 1,01 | 1,65 | *0,040* | 1,21 | 0,95 | 1,55 | *0,118* |
| **Child Characteristics** |  |  |  |  |  |  |  |  |  |  |  |  |
| **Sex of the Child** |  |  |  |  |  |  |  |  |  |  |  |  |
| Female | 1,00 |  |  |  |  |  |  |  | 1,00 |  |  |  |
| Male | 1,04 | 0,86 | 1,25 | *0,704* | 1,04 | 0,87 | 1,25 | *0,673* | 1,13 | 0,93 | 1,37 | *0,221* |
| **Combined birth rank and birth interval** |  |  |  |  |  |  |  |  |  |  |  |  |
| 2nd/3rd birth rank, more than 2-years interval | 1,00 |  |  |  | 1,00 |  |  |  | 1,00 |  |  |  |
| 1st birth rank | 1,52 | 1,22 | 1,88 | *<0,001* | 1,46 | 1,19 | 1,81 | *<0,001* | 1,45 | 1,18 | 1,79 | *<0,001* |
| 2nd/3rd birth rank, less than or equal 2-years interval | 0,78 | 0,52 | 1,18 | *0,237* | 0,87 | 0,58 | 1,30 | *0,501* | 0,71 | 0,48 | 1,05 | *0,089* |
| 4th birth rank, more than 2-years interval | 1,13 | 0,84 | 1,52 | *0,405* | 0,80 | 0,60 | 1,06 | *0,121* | 1,20 | 0,90 | 1,59 | *0,215* |
| 4th birth rank, less than or equal to 2-years interval | 0,63 | 0,35 | 1,14 | *0,125* | 0,55 | 0,31 | 0,97 | *0,038* | 0,97 | 0,55 | 1,71 | *0,918* |
| **Child's size at birth** |  |  |  |  |  |  |  |  |  |  |  |  |
| Average | 1,00 |  |  |  | 1,00 |  |  |  | 1,00 |  |  |  |
| Smaller than average | 1,35 | 1,03 | 1,78 | *0,031* | 1,15 | 0,84 | 1,55 | *0,383* | 1,13 | 0,84 | 1,52 | *0,419* |
| Larger than average | 0,97 | 0,79 | 1,20 | *0,802* | 0,93 | 0,76 | 1,15 | *0,516* | 0,89 | 0,72 | 1,10 | *0,274* |
| **Age of pregnancy** |  |  |  |  |  |  |  |  |  |  |  |  |
| Term | 1,00 |  |  |  | 1,00 |  |  |  | 1,00 |  |  |  |
| Preterm | 0,87 | 0,49 | 1,53 | *0,621* | 1,46 | 0,85 | 2,53 | *0,173* | 1,40 | 0,82 | 2,38 | *0,217* |
